# Bovine Serum Albumin-Immobilized Black Phosphorus-Based γ-Fe_2_O_3_ Nanocomposites: A Promising Biocompatible Nanoplatform

**DOI:** 10.3390/biomedicines9080858

**Published:** 2021-07-22

**Authors:** Atanu Naskar, Sohee Lee, Dongjoon Ko, Semi Kim, Kwang-sun Kim

**Affiliations:** 1 Department of Chemistry and Chemistry, Institute for Functional Materials, Pusan National University, Busan 46241, Korea; atanunaskar@pusan.ac.kr (A.N.); kin5497170@pusan.ac.kr (S.L.); 2 Immunotherapy Research Center, Korea Research Institute of Bioscience and Biotechnology, Daejeon 34141, Korea; kdj8915@kribb.re.kr (D.K.); semikim@kribb.re.kr (S.K.)

**Keywords:** black phosphorus nanocomposite, nanomaterial–protein interaction, serum albumin protein, iron oxide nanocomposite, nanoplatform

## Abstract

The interactions between proteins and nanoparticles need to be fully characterized as the immobilization of proteins onto various nanoplatforms in the physiological system often results in the change of surface of the protein molecules to avoid any detrimental issues related to their biomedical applications. Hence, in this article, the successful low-temperature synthesis of a BP-based γ-Fe_2_O_3_ (IB) nanocomposite and its interactive behavior with bovine serum albumin (BSA)—a molecule with chemical similarity and high sequence identity to human serum albumin—are described. To confirm the formation of γ-Fe_2_O_3_ and the IB nanocomposite, X-ray diffraction, transmission electron microscopy, and X-ray photoelectron spectroscopy analyses of the materials were performed. Additionally, the physical interaction between BSA and the IB nanocomposite was confirmed via UV–Vis and photoluminescence spectral analyses. Finally, the biocompatibility of the BSA-immobilized IB nanocomposite was verified using an in vitro cytotoxicity assay with HCT-15 colon cancer cells. Our findings demonstrate that this newly developed nanocomposite has potential utility as a biocompatible nanoplatform for various biomedical applications.

## 1. Introduction

The use of nanoparticles in various biomedical applications has developed rapidly and successfully in recent years [1,2]. The unique physicochemical properties of nanomaterials, such as their high surface area-to-volume ratio and specific size-dependent properties, have facilitated their use in drug delivery, bioimaging, antibacterial, biosensing, and other applications [3,4]. In this regard, various polymeric and inorganic nanomaterial have been employed for biomedical applications such as gene silencing, gene therapy, drug delivery, gene delivery, bioimaging, and others [5,6,7,8,9,10,11,12,13].

Notably, whenever any nanomaterial enters a physiological environment or biofluid, formation of a protein “corona” ensues; that is, the surface of the nanomaterial is covered with layers of proteins via physicochemical interactions [14]. This protein corona not only results in changes to the structure and function of the proteins but also alters the synthetic nanomaterial to a more biological one [15]. As such uncontrolled nanomaterial–protein interactions may hinder the efficient removal of the nanoparticles from the body through enzymatic cascades, fatal consequences may occur from their direct presence in the blood circulatory system [16]. Hence, controlling the interaction of nanoparticles with biological interfaces, such as proteins, is a critical challenge in biomedical applications. Nanomaterials can be used in biomedical applications only when their safety (i.e., non-toxicity), effectiveness, and physiological responses can be fully controlled. In particular, the effective control of the protein–nanoparticle interactions and a more detailed understanding of these interactions may help to reduce the potential risk of nanomaterials in biomedical applications.

Among the various nanomaterials used for biomedical purposes, metal oxide nanoparticles have received much attention [17,18]. For example, maghemite (γ-Fe_2_O_3_; hereinafter abbreviated as IO) is often used for its anticancer activity and for drug delivery, bioimaging, and biosensing applications [19,20]. Additionally, with the immense success of graphene, the use of a wide range of thin 2D materials in biomedical products is on the rise [21]. In this aspect, boron nitride, graphite carbon nitride, MXenes, and black phosphorus (BP) were preferred because of their unique physicochemical and biosafety profiles. Among these, BP has been recently used in anticancer, antibacterial, biosensing, drug delivery, and other biomedical applications [18,20,22]. The thermodynamic stability of BP at ambient temperature, as well as its non-toxicity, wide band gap range (0.3–2.0 eV), broad absorption property, and ambipolar characteristic make it an ideal candidate for use in biomedicines [22,23]. However, despite the enormous biomedical potential of IO and BP, there is limited knowledge of the protein interactions that occur in BP-based IO (hereinafter abbreviated as IB) nanocomposites.

The three-dimensional structures of proteins, which include multi-level conformations, are highly linked to their biological functions and immune response activation [24]. As a result, it is easy to recognize how the interaction of nanomaterials with proteins affects the structure and normal functions of proteins. Furthermore, covalently conjugated proteins encapsulated by nanomaterials significantly increased stability of proteins. Therefore, the protein–corona formation or the interaction between nanomaterials and proteins is required to understand the role of nanomaterials on protein-associated physiology. Serum albumin is a water-soluble protein that plays an important role in the transportation of biomaterials, including fatty acids, amino acids, steroids, and a variety of drugs [14,15]. It is the most abundant protein in the blood plasma of the circulatory system, with various physiological functions [15]. Therefore, the elucidation of the interactions between serum proteins and nanomaterials is of utmost importance before the latter can be used for in vivo applications. Bovine serum albumin (BSA), a heart-shaped globular protein, has been widely used as an ideal model for studying the interactions between human serum albumin (HSA) and nanomaterials, as it shares sequence and structural similarities (76%) with its human protein counterpart [25]. Studies on the interactions of BSA with Ag [26], Au [27], ZnO [14], CdS [28], TiO_2_ [29], ZnO-graphene [14], and Au-ZnO-graphene [25] have provided important insights into the protein–nanomaterial interactions. However, the interactions between BSA molecules and BP-based nanoplatforms and the biological responses to such nanocomposites, as well as their cytotoxicity, have still not been investigated.

This study aimed to synthesize an IB nanocomposite and elucidate its interactions with BSA using fluorescence and absorption spectroscopy techniques. Additionally, the effect of the BSA-immobilized IB nanocomposite (hereinafter designated as the IBB nanocomposite) on cell proliferation was analyzed to verify its biocompatibility for biomedical applications.

## 2. Materials and Methods

### 2.1. Synthesis of γ-Fe_2_O_3_

For the preparation of the IO particles [30], 0.05 M ferric nitrate nonahydrate (1.0085 g; Fe(NO_3_)_3_∙9H_2_O; 98% purity; Merck, MA, USA) was uniformly dissolved in 50 mL of deionized water by continuous stirring for 30 min. Then, 1 mL of hydrazine hydrate (H_6_N_2_O, 99–100% purity; Merck, MA, USA) was added dropwise to the precursor solution, and the mixture was ultrasonicated for 10 min in a water bath ultrasonicator. During this process, some precipitation of particles can be seen at the bottom of the solution. Meanwhile, the pH was checked regularly during the entire process, and the same procedure was repeated until the solution pH reached a value of 9. Thereafter, the IO-containing precipitate was separated by centrifugation (800 rpm, 5 min) and thoroughly washed with deionized water and ethanol. Finally, the IO sample was dried in an air oven at 60 °C for 8 h.

### 2.2. Synthesis of Various Nanocomposites

#### 2.2.1. Synthesis of Black Phosphorus Nanosheets

BP nanosheets were synthesized using the methods described in our previous work [20].

#### 2.2.2. Synthesis of Black Phosphorus-Based γ-Fe_2_O_3_ Nanocomposites

In brief, 0.1 g of the as-prepared IO nanomaterial and 2 mL (2 mg × mL^−1^) of the as-prepared BP nanosheets were dispersed in 30 mL of deionized water for preparation of the IB nanocomposite. After 30 min of ultrasonication, the mixture was stirred continuously for 6 h. Then, the nanocomposite product was separated by centrifugation and washed with deionized water and ethanol. Finally, the sample was collected after vacuum drying at 60 °C for 6 h.

### 2.3. Synthesis of BSA-Immobilized Black Phosphorus-Based γ-Fe_2_O_3_ Nanocomposites

To obtain the IBB nanocomposite, a procedure similar to that described in our previous study was used [25]. In brief, a 10^−6^ M aqueous solution of BSA (≥ 94–98% purity, Sigma-Aldrich, St. Louis, MO, USA) was firstly prepared. Then, 20 mL of the BSA solution was mixed with 20 mg of the IB nanocomposites, and the mixture was ultrasonicated for 1 h. Thereafter, the mixture was stirred overnight at ambient temperature (21–25 °C) to allow complete interaction between the IB nanocomposite and BSA molecules. The IBB nanocomposites were then collected by centrifugation (8000 rpm, 3 min) and washed with deionized water and ethanol to remove free BSA molecules. Finally, the IBB nanocomposites were oven dried and stored for further study.

### 2.4. Characterization of the Fabricated Nanomaterials

#### 2.4.1. Properties of the Nanomaterials

The diffraction patterns and structures of the IO and IB samples were assessed via X-ray diffraction (XRD) in the 2θ range of 5–80°, using a D8 ADVANCE X-ray diffraction unit with DAVINCI design (Bruker, Billerica, MA, USA) that was connected to a nickel filtered Cu K_α_ radiation source (λ = 1.5406 Å). Additionally, the microstructure of the IB nanocomposite was analyzed using transmission electron microscopy (TEM; Bruker Nano GmbH, Berlin, Germany), with the samples placed onto carbon-coated 300-mesh Cu grids. Furthermore, the chemical state of elements of the representative IB sample was explored using an Axis Supra Scanning X-ray photoelectron spectroscopy (XPS) microprobe surface analysis system. The binding energy range was set at 200–1200 eV. The C 1*s* peak position at 284.5 eV was used as the binding energy reference.

#### 2.4.2. Study of the IB Nanocomposite–BSA Interactions

Ultraviolet-visible (UV–Vis) spectroscopy was used to determine the interactions between the IB nanocomposite and BSA molecules. First, a 10^−6^ M aqueous solution of BSA was prepared in deionized water and stirred overnight. Subsequently, three different amounts (100, 500, and 1000 μg) of the IB nanocomposite were dispersed in 10 mL of the aqueous BSA suspension separately. Afterwards, the mixtures (designated as A, B, and C, respectively) were homogenized through ultrasonication for 30 min and then stirred with a magnetic stirrer for approximately 1 h. A UV–Vis spectrophotometer (lambda 465; PerkinElmer, Waltham, MA, USA) was used to measure the UV–Vis absorption spectra of solutions A, B, and C in the wavelength range of 200–650 nm. Additionally, the photoluminescence spectra of solutions A, B, and C were recorded with a fluorescence spectrometer (FL 6500; PerkinElmer, Waltham, MA, USA) to further analyze the interactions between the IB nanocomposite and BSA molecules. The emission spectra were measured in the wavelength range of 300–425 nm, with an excitation wavelength of 278 nm.

#### 2.4.3. WST Assay for Determining Cell Proliferation

A WST assay kit (Ez-Cytox; Dogenbio, Seoul, Korea) was used to determine the cytotoxicity of the IB and IBB nanocomposites. The HCT-15 colon cancer cells used for this study were purchased from the American Type Culture Collection (Manassas, VA, USA) and maintained in RPMI 1640 medium, supplemented with 10% fetal bovine serum, at 37 °C in 5% CO_2_. For the assay, 4000 cells were seeded into each well of a 96-well plate and incubated for 24 h. Then, the cells were further incubated in the presence of the IO, IB, or IBB sample (at concentrations of 10–200 µg × mL^−1^ in 0.1% dimethyl sulfoxide) for 24 or 48 h. Thereafter, the cells were incubated with the WST reagent (one-tenth of the medium volume), and the amount of formazan dye formed was determined by measuring the absorbance at 450 nm using a spectrophotometric microplate reader (BMG LABTECH GmbH, Ortenber, Germany).

## 3. Results and Discussion

### 3.1. Properties of the Materials

#### 3.1.1. XRD Analysis

**Figure 1:**
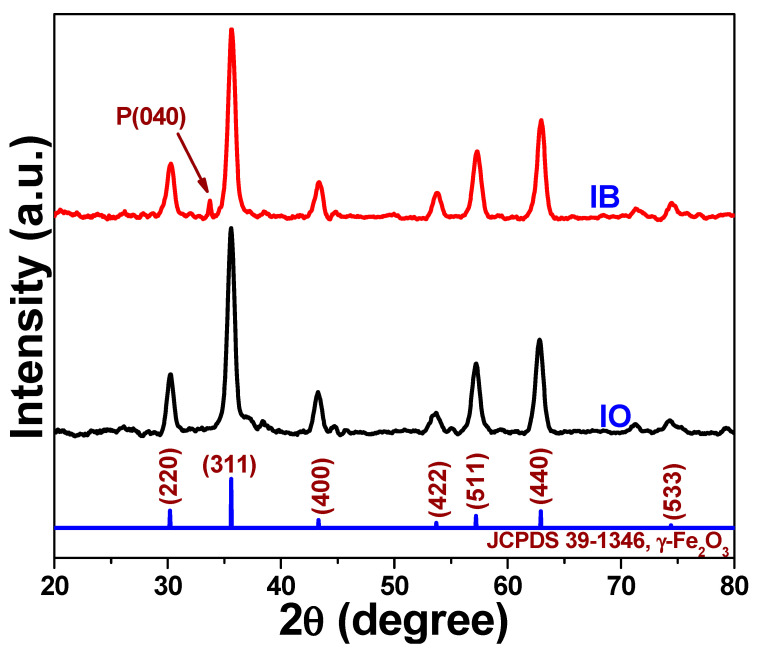
X-ray diffraction patterns of the γ-Fe_2_O_3_ (IO) and black phosphorus-based γ-Fe_2_O_3_ (IB) samples.

The crystallinity of the synthesized IO and IB products was analyzed with XRD (Figure 1). The XRD peaks of the two samples were found to match entirely with the spinel structure of γ-Fe_2_O_3_ (JCPDS 39-1346) [20]. However, in the IB sample, one additional peak was observed at 33.80°, which corresponded to the (040) lattice plane of BP [20]. This result confirmed the orthorhombic nature of the exfoliated BP nanosheets. Moreover, no impurity peaks were evident, even after liquid ultrasonication. Therefore, the successful synthesis of the IB nanocomposite was effectively verified by the XRD data (Figure 1).

#### 3.1.2. Morphology and Microstructure of the Fabricated Products

**Figure 2:**
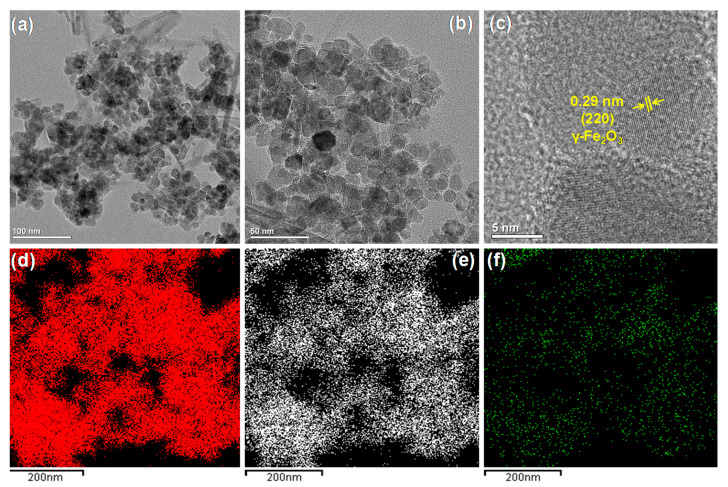
(**a**,**b**) Transmission electron microscopy (TEM) images and (**c**) high-resolution TEM image of the IB sample, with elemental mappings of (**d**) Fe, (**e**) O, and (**f**) P.

Figure 2 shows TEM images of the surface morphology and elemental mapping results of the representative IB nanocomposite. Figure 2a,b show TEM images of the IB microstructure, where the shape of the nanoparticles was confirmed to be quasi-spherical. Additionally, the IO particles were found to be well dispersed within the BP layers. The corresponding high-resolution TEM image (Figure 2c) of the IB sample showed distinct lattice fringes with an interplanar distance of 0.29 nm, which corresponded to the (220) plane of γ-Fe_2_O_3_ [20]. Elemental mapping of the IB sample revealed the existence of Fe (Figure 2d), O (Figure 2e), and P elements (Figure 2f). These observations supported the XRD results (Figure 1), thereby confirming the formation of the IB nanocomposites.

#### 3.1.3. X-ray Photoelectron Spectra

**Figure 3:**
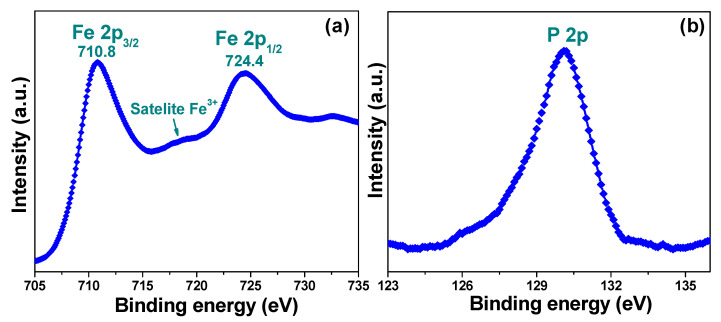
X-ray photoelectron spectra of the binding energies of the (**a**) Fe 2*p* and (**b**) P 2*p* core levels of the IB nanocomposite.

Figure 3 depicts the X-ray photoelectron spectra of the representative IB sample, which were measured to determine the surface chemical composition and valence state of the constituent elements of the nanocomposite. The binding energy signals of the Fe *2p* and P *2p* peaks are shown in Figure 3a and Figure 3b, respectively. For Fe *2p* (Figure 3a), the two strong peaks observed at 710.8 and 724.4 eV were assigned to the binding energies of the Fe *2p*_3/2_ and Fe *2p*_1/2_ states, respectively [20]. It is worth noting that these are the representative peaks of Fe^3+^. A satellite peak was also seen at approximately 719 eV, which further confirmed the formation of γ-Fe_2_O_3_, as this satellite peak is characteristic of the Fe^3+^ ion in this metal oxide [20]. Additionally, the presence of the P *2p* peak (Figure 3b) further confirmed the formation of the IB nanocomposite.

### 3.2. IB Nanocomposite–BSA Interaction Studies

#### 3.2.1. Ultraviolet-Visible Spectra

**Figure 4:**
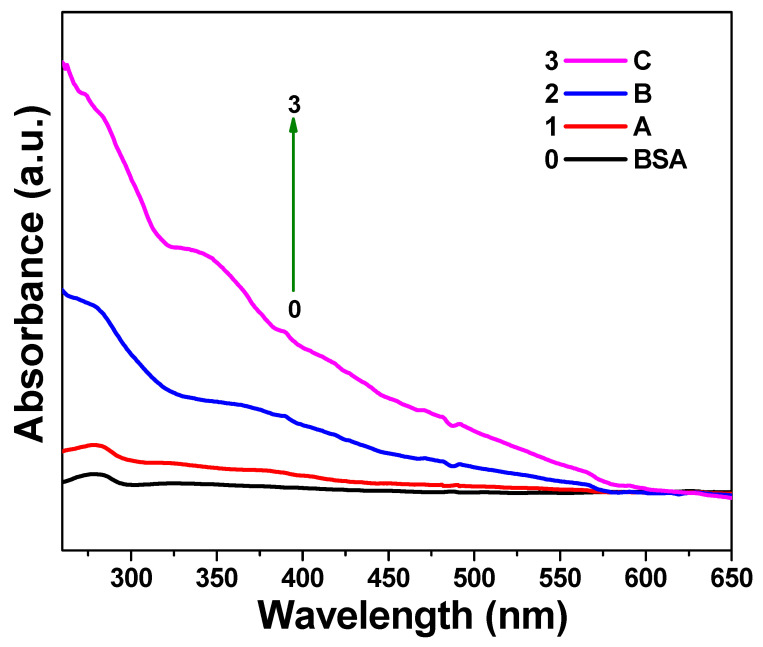
UV–Vis absorption spectra of aqueous bovine serum albumin (BSA) (10^−6^ M) solutions in the presence of varying IB amounts: 100 µg (**A**), 500 µg (**B**), and 1,000 µg (**C**) in 10 mL of aqueous BSA solution.

The UV–Vis absorption spectra of BSA and the A, B, and C solutions were measured (Figure 4) to study the interactions between the protein and IB nanocomposite. There was an obvious absorption peak at approximately 280 nm in the solution with BSA only [14]. However, the peak intensity appeared to increase gradually with increasing IB concentrations, as evidenced from the A, B, and C solution peaks. The peak intensity of the BSA solution increased according to the increasing concentration of the IB nanocomposite; that is, C > B > A. Hence, it could be surmised that the enhancement in absorption peak intensity at approximately 278 nm was due to the addition of increasing concentrations of the IB nanocomposite. These results confirmed the formation of the IBB nanocomposite (i.e., BSA in complex with IB).

#### 3.2.2. Photoluminescence Spectra

**Figure 5:**
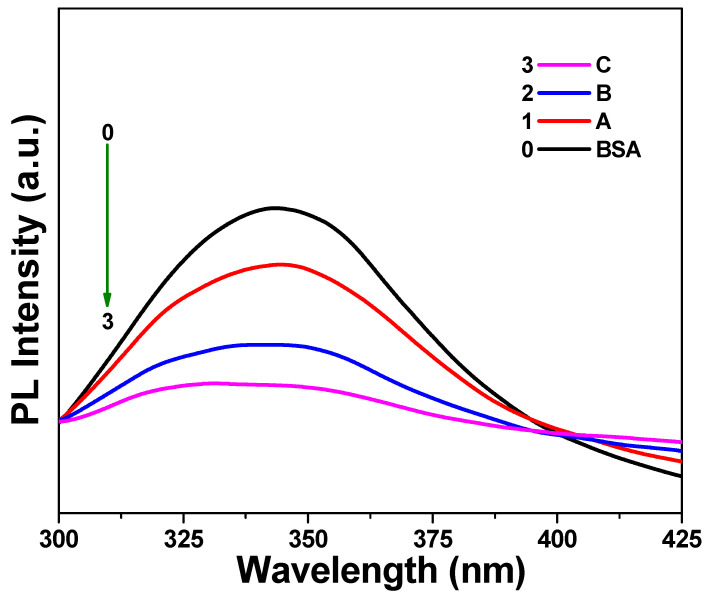
Photoluminescence (PL) spectra of aqueous BSA (10^−6^ M) solutions in the presence of varying IB amounts: 100 µg (**A**), 500 µg (**B**), and 1000 µg (**C**) in 10 mL of aqueous BSA solution.

In a further approach, a photoluminescence spectral study was carried out to evaluate the interactions between BSA and the IB nanocomposite. Similar to the UV–Vis spectral studies, the photoluminescence spectra of BSA in the absence and presence of different IB concentrations were recorded. As shown in Figure 5, an emission band could be clearly seen at approximately 350 nm for the BSA solution only when excited at 278 nm wavelength (Figure 4) [14]. However, contrary to the UV–Vis spectral peaks, the relative intensity of the photoluminescence peaks for solutions A, B, and C decreased gradually; that is, A > B > C. These results imply that an increase in IB nanocomposite concentration causes a decrease in the photoluminescence peak intensity of BSA. Furthermore, the photoluminescence peak observed at approximately 350 nm was found to have shifted toward a higher wavelength region with an increase in IB concentration [25]. These results strongly suggest an interaction between the BSA molecules and the IB nanocomposite.

### 3.3. In Vitro Cytotoxicity of the Fabricated Nanomaterials

**Figure 6:**
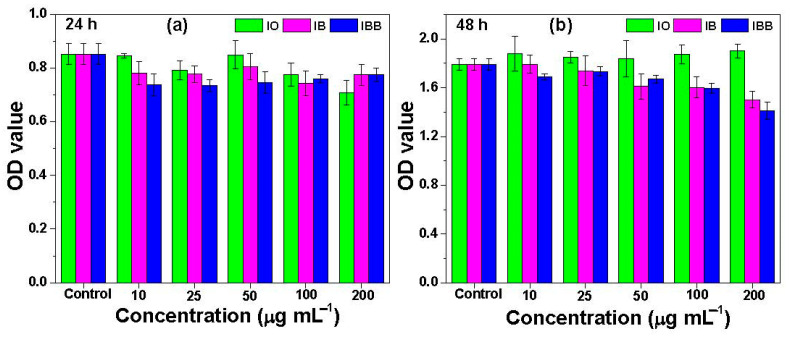
WST assay determination of the viability of HCT-15 colon cancer cells in the presence of various concentrations of IO, IB, and BSA-immobilized IB (IBB) nanocomposites for (**a**) 24 h or (**b**) 48 h. The error bars represent the standard deviation (*p* < 0.05).

The WST assay (Figure 6) was used to determine the cytotoxic effects of varying concentrations of the fabricated nanomaterials on HCT-15 colon cancer cells. Each bar graph in the figure represents triplicate measurements of the samples. It was clear from the results that the HCT-15 cells showed excellent viability on the IBB nanocomposite, even in the presence of 200 µg × mL^−1^ of the material. Therefore, it can be concluded that the BSA interaction with the IB nanocomposite is not harmful to human cells, and the IBB nanocomposite can be used for biomedical applications without concern over its cytotoxicity.

## 4. Conclusions

In summary, the interactions of a protein (BSA) with a BP-based γ-Fe_2_O_3_ nanocomposite and the effects of the fabricated nanomaterials on human cells were studied for the first time. A low-temperature process was used to synthesize the γ-Fe_2_O_3_ nanoparticles. Furthermore, the strong interactions between BSA and the IB nanocomposite were studied using UV–Vis and photoluminescence spectroscopy. The final IBB nanocomposite showed excellent biocompatibility with HCT-15 colon cancer cells. Therefore, this IBB nanocomposite is suitable for use as a novel nanoplatform for various biomedical applications. Additionally, our fabricated nanocomposites provide an avenue for the interaction studies of other proteins (e.g., HSA) in the future.

## Data Availability

Not Applicable.
